# Use of X-Ray Fluorescence Microscopy for Studies on Research Models of Hepatocellular Carcinoma

**DOI:** 10.3389/fpubh.2021.711506

**Published:** 2021-08-20

**Authors:** Tatjana Paunesku, Andrew C. Gordon, Sarah White, Kathleen Harris, Olga Antipova, Evan Maxey, Stefan Vogt, Anthony Smith, Luiza Daddario, Daniele Procissi, Andrew Larson, Gayle E. Woloschak

**Affiliations:** ^1^Radiation Oncology Department, Feinberg School of Medicine, Northwestern University, Chicago, IL, United States; ^2^Radiology Department, Feinberg School of Medicine, Northwestern University, Chicago, IL, United States; ^3^Department of Radiology, Division of Interventional Radiology, Medical College of Wisconsin, Milwaukee, WI, United States; ^4^X-Ray Sciences Division, Advanced Photon Source, Argonne National Laboratory, Argonne, IL, United States

**Keywords:** radioembolization of liver malignancies, X-ray fluorescence microscopy, rabbit (Lagomorph), yttrium 90 microspheres, hepatocellular carcinoma

## Abstract

**Introduction:** TheraSphere^®^ microspheres containing yttrium ^90^Y are among many radioembolization agents used clinically to reduce liver tumor burden, and their effects on cancer volume reduction are well-established. At the same time, concerns about off target tissue injury often limit their use. Deeper investigation into tissue distribution and long-term impact of these microspheres could inform us about additional ways to use them in practice.

**Methods:** Healthy rat liver and rabbit liver tumor samples from animals treated with TheraSpheres were sectioned and their elemental maps were generated by X-ray fluorescence microscopy (XFM) at the Advanced Photon Source (APS) synchrotron at Argonne National Laboratory (ANL).

**Results:** Elemental imaging allowed us to identify the presence and distribution of TheraSpheres in animal tissues without the need for additional sample manipulation or staining. Ionizing radiation produced by ^90^Y radioactive contaminants present in these microspheres makes processing TheraSphere treated samples complex. Accumulation of microspheres in macrophages was observed.

**Conclusions:** This is the first study that used XFM to evaluate the location of microspheres and radionuclides in animal liver and tumor samples introduced through radioembolization. XFM has shown promise in expanding our understanding of radioembolization and could be used for investigation of human patient samples in the future.

## Introduction

Cancers affecting liver tissue are among the most difficult to treat, regardless of their origin. Primary liver cancers account for more than 700,000 deaths per year worldwide and show an annual 2% increase among causes of mortality over the last 13 years (1). As much as 70–90% of primary liver cancer cases are attributed to hepatocellular carcinoma (HCC) (2). In cases when surgical resection is impossible, HCC treatments include the use of “small molecules” such as sorafenib (3) or external beam radiation such as stereotactic body radiotherapy (SBRT) (4). While the liver blood supply is primarily venous, the HCC blood supply is arterial (5) and this peculiarity allows use of intra-arterial HCC therapies such as chemoembolization and radioembolization (6–9). Each one of these treatments also has its own set of possible complications. Sorafenib, for example, can cause skin toxicity that is severe enough to necessitate dose reduction or discontinuation of therapy (10). Radiotoxicity can be an outcome of either external beam radiotherapy or radioembolization and careful calculation of allowable doses makes up a part of any treatment regimen (6, 11). Despite the fact that radiopharmaceutical therapy is generally considered effective for treatment of many types of cancer (12), liver cancer radioembolization is discontinued just prior to treatment in as many as one third of all patients due to a variety of concerns (13). New approaches for safer radioembolization or combination treatments are needed. To this end, detailed evaluation in animal models of liver cancer treatments needs to be developed beyond current capabilities.

In order to investigate the feasibility of different treatment combinations and approaches, the use of animal models is extremely important for evaluation of radioembolization. Among them, the rabbit VX2 liver cancer model is one of the most valuable as the size of these animals permits use of diagnostic and treatment approaches similar to those used in human patients. Clinically used agents for chemoembolization (5) and radioembolization (14) were explored in this model for a long while. More recently, this model is used for testing of novel therapeutic and diagnostic agents such as nanoparticles (15–19) and targeted nanoparticles (20, 21), both alone and in combination with radionuclides (22–24). VX2 cells are a rabbit cell line, originally induced by cottontail rabbit papilloma virus (25), that grows well in immunologically competent animals. The tumors generated by VX2 implantation are well-vascularized and, in liver, similar to HCC. We have used this animal model for different types of studies for more than 15 years (14, 20, 23, 26–31). Over the same period, we have also worked on introducing a unique imaging technique, x-ray fluorescence microscopy (XFM), into pathology (20, 32–36). Although we have previously used XFM to image liver VX2 samples (20), this is the very first study where we have developed and implemented necessary sample preparation techniques to process radioactive tissue samples and make them suitable for XFM imaging. In this work, we were able to identify individual TheraSpheres used for radioembolization both by their silica and yttrium content. At the same time, we could see the tissue and cell outlines through their biological element content, as 2D features in phosphorus, sulfur, iron, zinc etc. This approach has great promise for evaluation of processes ongoing in tissues containing radionuclides. Due to natural elemental content, XFM maps of biological samples are analogous to images of hematoxylin-and-eosin (H&E) stained samples, while radionuclides and other inorganic materials present in tissues can be registered and quantified.

## Methods

### Animals

This study used New Zealand white rabbits weighing between 12 and 20 lbs, used as either donor rabbits or liver-cancer animals (with VX2 tumors implanted into the liver under ultrasound guidance). The institutional animal care and use committee of Northwestern University approved all work in this study. As mentioned in the introduction, our work with VX2 rabbits has been extensive over the past 15 years, in different studies we used VX2 tumor bearing rabbits for a wide variety of endpoints (6, 14, 20, 23, 26–31).

VX2 cells, originally procured from National Cancer Institute (NCI, Frederick, MD, USA) were injected in the hind limb of a donor rabbit and allowed to grow for 3–4 weeks. Tumor growth was checked by palpation and the donor animals were sacrificed when the tumors reached 2–3 cm. These tumors were excised and dissected, and the viable tumor tissue was then cut into small sections and suspended in sterile Hank's solution (Sigma).

Recipient liver cancer rabbits were anesthetized by intramuscular injection of ketamine at 44 mg/kg and xylazine 3–5 mg/kg, and the rabbit was maintained under inhalational isoflurane at 2–3% during the procedure. Rabbit's abdomen was shaved and a preliminary ultrasound (Mindray M7, Midray Medical Intl Ltd.) was done with a L14-6S transducer. A millimeter long incision in the skin above liver was done under aseptic conditions and a coaxial introducer was inserted into the liver under direct ultrasound guidance. Several small tumor fragments were pushed through the introducer into the liver and a final ultrasound was performed to assess for complications including bleeding. The anesthetic was reversed with yohimbine 0.5 mg/kg (Lloyd Laboratories). Meloxicam 0.2 mg/kg (Norbrook Laboratories Ltd., Newry, Northern Ireland) was administered for pain. After tumor implantation procedure the rabbits were monitored daily for pain, lethargy, appetite and mobility. Two weeks after surgery, the tumor growth was monitored using 7T magnetic resonance imaging. This work followed procedures used routinely in the past (14, 26–31, 37).

### Radioembolization

For these experiments we used clinical microspheres TheraSphere^®^ (BTG Interventional Medicine) containing yttrium ^90^Y. The beads were made of glass, 20–30 μm in diameter with specific gravity of 3.6 g/cc. The use of glass ^90^Y microspheres for research was approved and monitored by the Radiation Safety Office of the Northwestern University.

Rabbit treatments with microspheres were done in animals with liver tumor growth confirmed by MRI imaging. Microspheres were injected using a microcatheter (Renegade HI-FLO, Boston Scientific) and microwire (Glidewire^®^ GT; 0.018", 180 cm) used under fluoroscopic guidance (OEC 9800 Plus mobile C-arm and vascular platform workstation, GE Medical Systems) in order to approach the left hepatic artery *via* celiac artery and femoral artery. Digital subtraction angiography (Omnipaque, GE Healthcare) was used to identify the anatomy and confirm the target treatment volume, native arterial flow, and reflux. Radioembolization was done with 9 mg of microspheres (~1 GBq) per animal, followed by 30–40 ml of sterile 0.9% saline over 3–5 min. Finally, the femoral artery was ligated and the animals were maintained for 2–3 more weeks depending on their health status. The rabbits were monitored daily for pain, lethargy, appetite and mobility.

TheraSpheres treatments of healthy animals were done with healthy Sprague-Dawley rats (Charles River Laboratories, Wilmington, MA), 450–500 g of weight. Catheterization of hepatic lobe in a rat required a surgical procedure where the animal's abdomen was opened, mesenteric venous drainage was used to select the right portal vein and a catheter was used to inject 9 mg of TheraSpheres and 6–8 ml of sterile 0.9% saline over 1–2 min. In this case partially decayed microspheres (day 5 after calibration to 1 GBq, corresponding to 0.2738GBq) were used. Ligation was done after infusion, the bowel was returned into the abdominal cavity, and the abdomen was closed in two layers. Animals were monitored daily and sacrificed 2 months after this procedure.

Administered activity on the day of animal treatment is provided in Table 1. While activity indicated in Table 1 came primarily from ^90^Y, several other radionuclides are also present in TheraSpheres. Some of them have a significantly longer half-life than ^90^Y (Table 2), and this generated additional concerns with sample processing and handling.

**Table 1:** Administered activity (primarily ^90^Y, for additional explanation see Table 2) on the day of animal treatment.

**Animal**	**Administered activity (Bq)**	**Date**
Rabbit 843382	80,586,899.58	10/25/15
Rabbit 827185	92,169,487.58	8/23/15
Rabbit 827186	69,696,624.87	8/23/15
Rabbit 762071	84,036,896.28	11/9/14
“Rat 21”	33,881,260.94	10/25/15

**Table 2:** MDS Nordion measurement of by-products at 60 days post-calibration for TheraSphere with Calibration dates after January 1, 2010.

**Isotope**	**Half-life**	**Energy line(s)**	**Average activity per unit mass**	
	**Days**	**keV**	**Bq/mg**	**SD (*n* = 13)**
Y-91	58.51	1,204.7	2,504	271
Y-88	106.65	898	895	64
Cr-51	27.7	320.1	78.9	9.1
Total for all other nuclides:			81	7.5

### Sample Preparation

At necropsy, the livers were separated into pieces suitable for freezing in molds with optimum cutting temperature solution (Tissue-Tek^®^ O.C.T. Compound, Sakura^®^ Finetek). Frozen samples were stored in a −80**°**C freezer and allowed to decay before further use.

Samples for X-ray fluorescence microscopy were prepared from livers of four rabbits (each with a VX2 tumor) and one rat, (example of “radio-surgery”) treated with TheraSpheres. Seven-micrometer-thick frozen tissue sections were prepared on a Leica cryostat dedicated to work with radioactive samples, placed on Ultralene membrane (SPEX Sample Prep, LLC, 15 Liberty St., Metuchen, NJ, USA) and allowed to air dry. Radioactivity of these samples was followed with a hand-held Geiger counter in order to monitor possible contamination during processing. Once dried, these samples were positioned on an in-house 3D printed PLA sample support, Ultralene membrane backing was glued onto the PLA frame and trimmed. Samples were wrapped with the Ultralene membrane and the entire assembly secured with Kapton tape (Kapton Tape Com.) in order to prevent potential radioactive contamination of X-ray fluorescence microscope.

### X-Ray Fluorescence Microscopy

Elemental mapping was done at the Advanced Photon Source Synchrotron at two different instruments: X-ray microprobe at Sector 2, beamline station 2ID-E; and with the “large area instrument” at the Sector 8 bending magnet beamline 8BM-B). At the large area instrument, at the beamline station 8BMB, KB mirror are used to obtain a 30 micron beam spot size for a high throughput overview of elemental distribution in tissues. Spectra were collected with a SII Vortex ME4 4-element silicon drift detector (SII NanoTechnology USA, Northridge, CA). For calibration we used thin film AXO standards (Applied X-ray Optics, Dresden, Germany) and the peaks were deconvoluted using MAPS software (38). Per pixel counts were converted to elemental concentrations (μg/cm^2^). Hard X-rays energy of 21 and 15 keV were both used for scanning at 8BM-B station. While K line of Y was imaged at 21 keV, scanning at 15 keV was also used in order to increase yield of fluorescent signals from biologically relevant elements. At this combination of energies all elemental components of interest were detected by their K alpha fluorescence. This included not only “native biological elements,” but also silica—major component of glass microspheres and yttrium itself.

For higher resolution scans at the Sector 2-ID-E samples were raster-scanned with a beam focused to 0.3 micron using Fresnel Zone Plates. At this station only hard X-rays of 21 keV were used. Silicon drift energy dispersive detector positioned was used to collect the fluorescence signal from samples, at 90° to the incident beam. Per pixel elemental concentration was obtained by comparison with the thin-film standards NBS-1832 and NBS-1833 from the National Bureau of Standards (Gaithersburg, MD), and the analysis was done using MAPS software (38) as detailed in other studies (20, 34–36).

## Results

### “Radio-Surgery” of Healthy Rat Liver

The distribution of TheraSpheres was investigated in four rabbit VX2 liver cancer samples and a single rat with tumor free liver, all exposed to TheraSpheres as a means of “radio-surgery” (see Table 1). One additional rabbit treated with cold TheraSpheres was included in the analysis (Supplementary Figure 1, obtained at station 2-ID-E with X-ray energy of 21 keV). This sample allowed us to see how much of the XFM yttrium signal can be ascribed to the “overflow” of the very strong Si signal. In samples treated with cold and hot TheraSpheres Si signal maxima were comparable (191 and 227 micrograms per square centimeter) while signal maxima for artifact Y signal vs. true Y signal differed 1,000-fold (0.56 vs. 515 micrograms per square centimeter).

**Figure 1:**
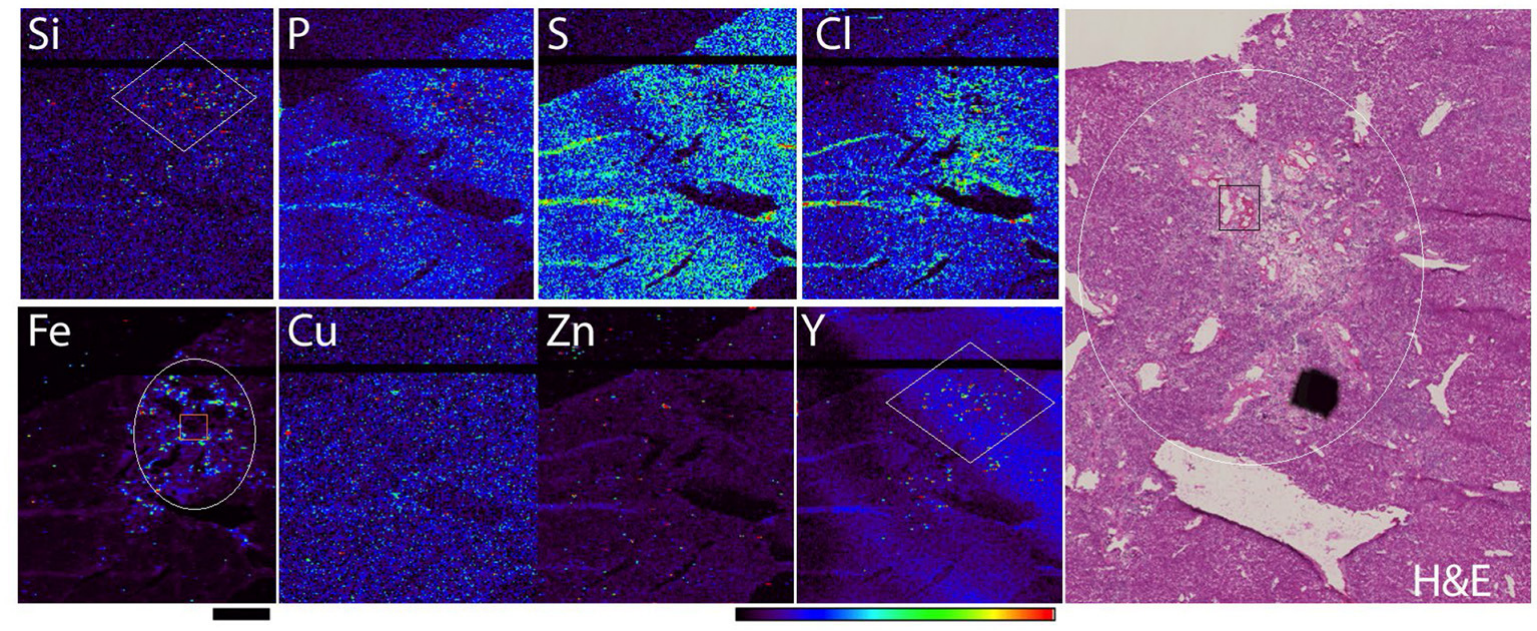
Rat liver tissue elemental map obtained with 30 micron spot size scanning, X-ray energy 15 keV (Station 8BM-B). Elemental maps were obtained simultaneously while sample was moved by rapid raster scanning (so called: “fly scans”) through the X-ray beam. Scale bar−2 mm, color bar indicates elemental quantity as represented by false colors, from black (no signal) to red (highest signal). Hematoxylin and eosin (H&E) staining of the same region in one of the adjacent tissue sections is shown in the right-hand panel. White diamonds in Si and Y maps correspond to a TheraSpheres rich area; white circle indicates fibrotic area in Fe map and H&E image. Orange rectangle in Fe image and black rectangle in H&E image correspond to areas shown in Figure 2 (due to imaging setup, in Figure 2 these areas are horizontally flipped).

For sample prep for XFM, tissues were frozen in optimum cutting temperature (O.C.T.), sectioned at 7 microns on a cryostat and placed onto an Ultralene membrane to be scanned by X ray fluorescence microscopy (XFM). XFM images of the rat liver were obtained with different X-ray energies. We first obtained a large overview image shown in Figure 1 with 15 keV energy X-rays at 8BM-B beamline (30 micron spot size). The XFM technique gives simultaneous maps for biological elements P, S, Cl, K, Ca, Mn, Fe, Cu, Zn, as well as elements that make the TheraSphere microparticles—Si and Y (in scans at 21 keV). It should be noted that the sample area with the highest Si signals (dots in red color which represents the highest signal) is outlined by a white diamond. This region of the sample also has the highest Fe signal. We have noticed in other tissue samples that accumulation of fibroblasts (fibrosis) is associated with an increase in iron accumulation. Fibrotic area in Fe signal and H&E images are outlined by white ovals. Weak staining in the necrotic area of the H&E image corresponds with the development of fibrosis in the same area of the sample. Subsequent high-resolution imaging of the same sample at 21 keV energy was done in a sub-area of this tissue, labeled in Figure 1 as described in the Figure 1 legend.

**Figure 2:**
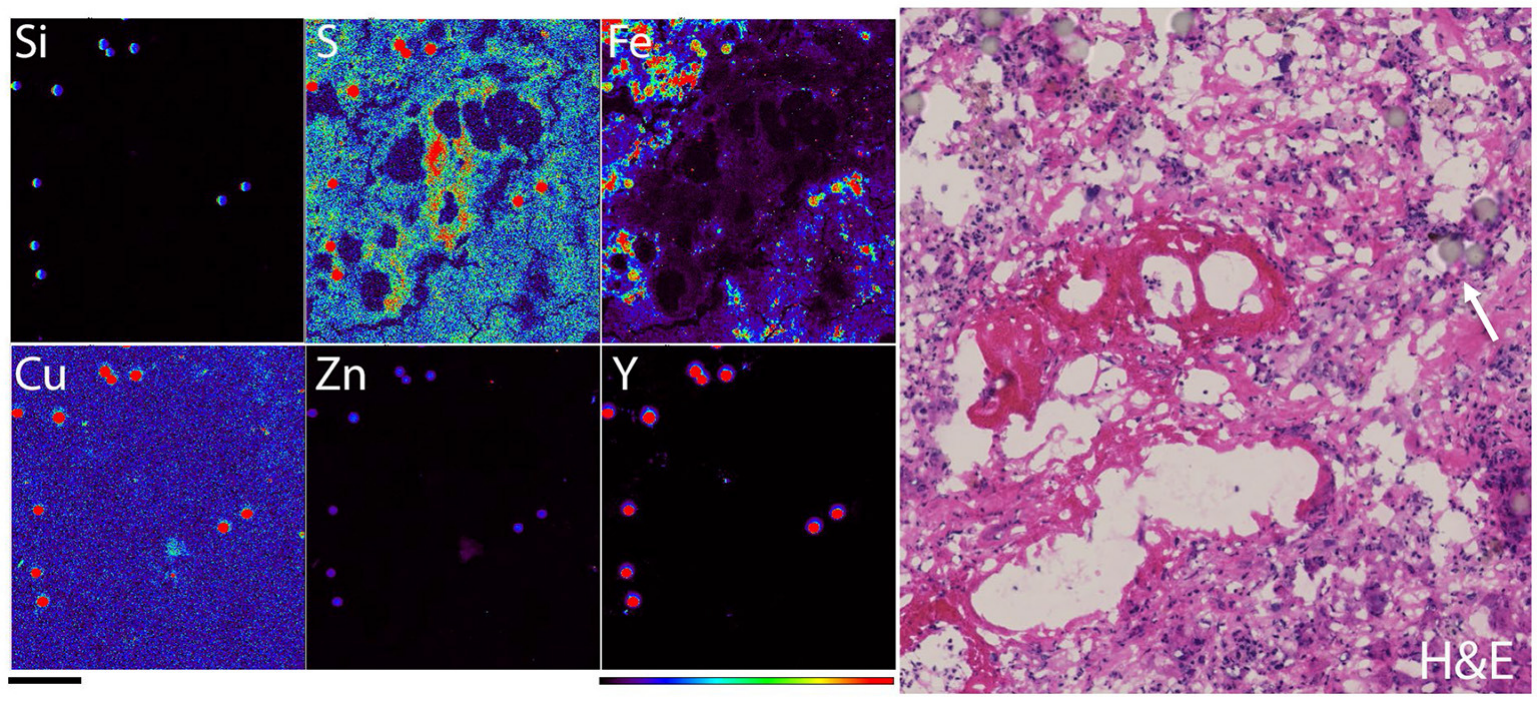
Elemental map of a detail of rat liver tissue obtained with 300 nanometer spot size (station 2ID-E), X-ray energy 21 keV. At this x-ray energy fluorescence produced by yttrium overwhelms signals of most biological elements and the speres “appear in all elemental channels.” Elemental maps were obtained simultaneously while sample was moved by rapid raster scanning (so called: “fly scans”) through the X-ray beam. Scale bar−200 microns, color bar indicates elemental quantity as represented by false colors, from black (no signal) to red (highest signal). Elemental signal maxima in micrograms per cm square for different elements were: 804 for Si, 14.5 for S, 0.499 for Cu, 37.1 for Fe, 7.79 for Zn and 774 for Y. Hematoxylin and eosin staining of the same region in one of the adjacent tissue sections is shown in the right hand panel; white arrow points to a TheraSphere. Please note that the same areas were scanned at lower magnification in Figure 1, as explained.

A closer look into the same area (Figure 2) was obtained by conducting XFM at the 2ID-E beamline where the sample was scanned with a beam size of 300 nm and at an X-ray energy of 21 keV (optimal for K alpha excitation of yttrium). Figure 2 shows a spread of microspheres (strong Si and Y signals of clearly spherical, 20–30 micron beads) surrounding an area with little iron and high sulfur signals, matching a region of necrotic changes where cell debris and extracellular matrix create a dense protein mesh (proteins are the major source of S in XFM images) with gradually accumulating fibrosis (Fe rich signal outside of the microsphere rich area). H&E image of the same sample region in one of the subsequent tissue sections shows the appearance of TheraSpreres in visible light images. Their identification is difficult and depends mostly on the appearance of the spherical shape itself.

**Figure 3:**
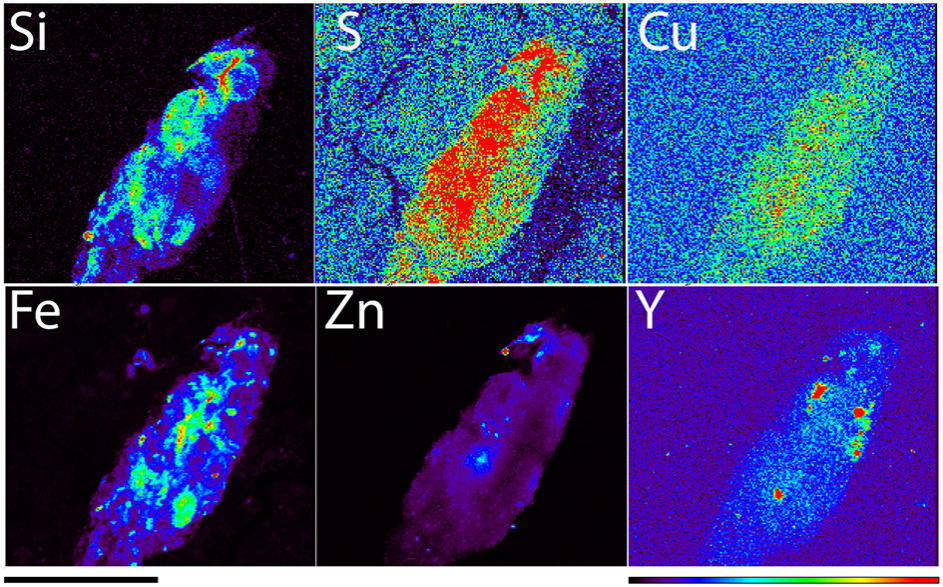
A detail of rat liver tissue elemental map obtained with 300 nanometer spot size (station 2ID-E), X-ray energy 21 keV. Scale bar −100 microns, color bar indicates elemental quantity as represented by false colors, from black (no signal) to red (highest signal). Signal maxima in micrograms per cm square for different elements were: 125 for Si, 5.45 for S, 0.199 for Cu, 50.9 for Fe, 23.7 for Zn, and 0.635 for Y.

In Figure 1 we can note that the microsphere rich area of the sample has the occasional “spots” with concentrated Zn signal that are independent from spots in Si and Y which correspond to microspheres. A closer investigation of one such Zn high region (Figure 3) has shown an accumulation of mostly “crushed” microspheres (although at least one found in the upper middle portion of the image retained its' spherical shape). The ratio of Zn and Fe in the same area of Figure 3 was an order of magnitude greater than Zn and Fe concentration in the overall region of microsphere spread (e.g., Figure 2). At the same time, concentration of yttrium within this region was smaller than in intact microspheres (Y maximum in Figure 2 in 774 vs. only 0.635 microgram per cm square in Figure 3). This suggests that the zinc rich structure in Figure 3 is a macrophage that has ingested several microspheres and damaged them structurally which led to a decreased Y concentration compared to Y maxima in intact microspheres.

### Radioembolization of VX2 Tumors in Rabbits

**Figure 4:**
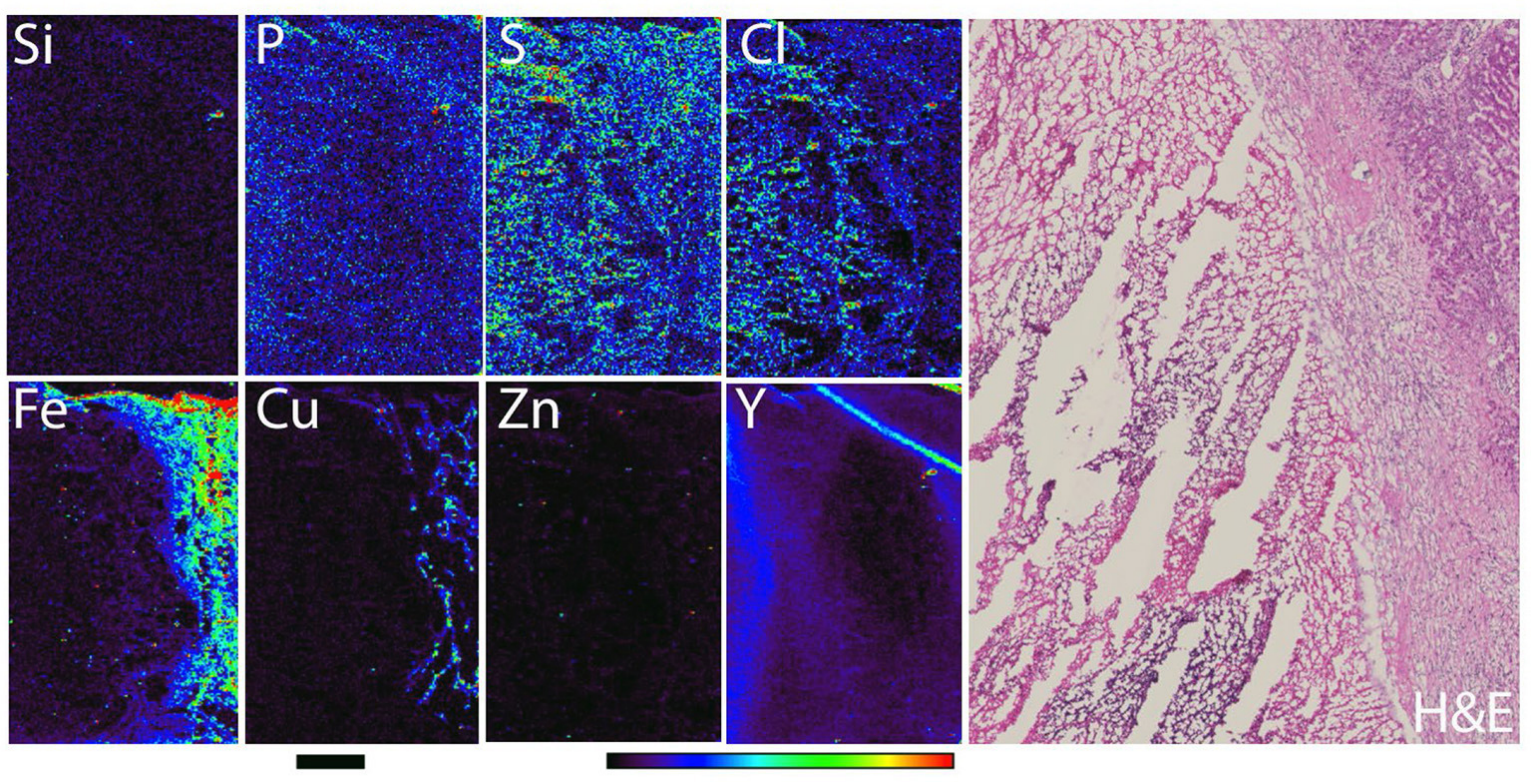
Rabbit liver with VX2 tumor necrotic region (left half of the sample) and VX2 viable part of the tumor (right-hand side of the sample). Elemental map was obtained with 30 micron spot size scanning, at X-ray energy 15 keV (station 8BM-B). Scale bar−2 mm, color bar indicates elemental quantity as represented by false colors, from black (no signal) to red (highest signal). Hematoxylin and eosin staining of the same region in one of the adjacent tissue sections is shown in the right hand panel.

In rabbit liver embolization experiments, the same quantity of miscrospheres (in terms of glass/silica content, although not with respect to ^90^Y, see Table 1) was injected as in rats. Therefore, much fewer microspheres were noted in rabbit liver samples compared to rat liver (Figure 4). A “lacey-like” tissue consistency of tumor tissue in rabbits was notable, elemental concentration for chlorine was the greatest in the same region of the sample. The most dynamic region of tumor growth and the region with the best vasculature was found at the border between tumor and healthy liver parenchyma. In this region of the tissue, the concentration of iron was the highest as well as the concentration of copper Cu. No comparative modulation of copper was seen in rat liver tissue and it is possible that this increase in Cu is driven by the presence of the tumor. It is known that Cu is one of the elements that shows intense redistribution during angiogenesis (39). Considering that VX2 tumors are hypervascular, it is possible that increased Cu signal corresponds to the area of the tissue with the most active growth of tumor blood vessels.

## Discussion and Conclusions

Use of XFM in investigation of biological samples has rapidly grown over the past decade with the development of third generation synchrotrons with beamlines and endstations dedicated to biological samples (34, 37, 40–45). While some of these capacities existed at the Advanced Photon Source since early 2,000 s, only recently could they be utilized for samples with large areas of interest such as pathology samples. This possibility opened after development of rapid scanning protocols and introduction of new detectors. While these new developments enabled tomographic imaging of single cells or whole small organisms such as zebrafish embryos (46–49), they have also allowed rapid screening of large sample areas. Today, the samples as large as few centimeters can be imaged at sub-micrometer resolution. In this work we used XFM to investigate animal liver tissue samples and evaluate elemental distribution of native biological elements as well as materials introduced into this organ by injection of TheraSpheres, glass microspheres containing radioactive ^90^Y for radioembolization and radio-lobectomy.

While different approaches can be used to follow distribution of TheraSpheres *in vivo* (6, 14), these methods lack the resolution necessary to establish micro-dosimetry in *ex vivo* samples. It is conceivable that different patterns of TheraSphere distribution could be correlated to different treatment outcomes. While cytotoxicity is the primary reason for use of TheraSpheres, it has been observed in some patient cases that the healthy liver remnant may initiate liver regeneration after TheraSphere treatment. If this type of beneficial development can be correlated with specific TheraSphere distribution patterns, this could be exploited to improve HCC care.

In addition, by using XFM to evaluate TheraSphere treated samples, it may be possible to monitor the process of neoangiogenesis by focusing on Cu accumulation and redistribution in viable regions of the tumor. This may be of particular importance for investigation of HCC because of its hypervascularity. In fact, tumor microvasculature density can be used as a predictor of recurrence in surgically treated patients (50). Because of HCC hypervascularity, therapies that target neoangiogenesis have long been considered as a good approach for treatment of HCC. For example, anti-angiogenesis coupled with the antiproliferative drug sorafenib, the current standard of care for HCC, inhibits the receptor tyrosine kinases vascular endothelial growth factor receptor 2 (VEGFR-2), platelet-derived growth factor receptor-beta 1/2 (PDGFR-β), and the kinase RAF (51). Use of XFM for screening of HCC samples would allow us to monitor the response to sorafenib or other anti-angiogenesis treatments. It should be noted that radioembolization is also often used in combination with sorafenib (8, 52) therefore, XFM investigation of HCC samples from such patients could be doubly interesting.

Finally, investigation of radio-lobectomy by XFM was also shown to be informative. Investigation of a liver sample from a TheraSphere treated but otherwise healthy, rat demonstrates elemental changes caused by necrosis and fibrotic changes in this organ caused by the cytotoxic effect of TheraSpheres. In addition, presence of broken microspheres leached from most of the yttrium in the rat sample suggest that macrophages may be capable of redistributing the TheraSpheres. In HCC treatment such involvement of the macrophages would be detrimental to therapy.

In conclusion, this is an early pilot study that established operating procedures for imaging of highly radioactive samples by XFM. Next, this work also documented that tissue elemental mapping at different energies (15 and 21 keV) provides informative data both about the biological elements and the TheraSpheres. Nevertheless, much more work is still needed in order to fully develop procedures for tomographic and high throughput imaging of these samples. Some of the work could be done with samples generated with cold TheraSpheres. For example, procedures for tomographic imaging of samples that contained only silica and approaches for precise quantification of silica would support the work with samples generated from tissues exposed to radioactive TheraSperes in the distant the past. On the other hand, addition of complementary approaches for imaging of radioactive samples such as use of high-resolution beta-microimagers (e.g., from Biospace Lab) or 3D autoradiography (53) would complement studies conducted with fresh samples prepared soon after treatments with radiolabeled TheraSpheres. In short—many possible avenues are opened for continuation of these studies and deeper evaluation of TheraSphere treatments.

## Data Availability Statement

The original contributions presented in the study are included in the article/Supplementary Material, further inquiries can be directed to the corresponding author/s.

## Ethics Statement

The animal study was reviewed and approved by Northwestern University Animal Care and Use Committee.

## Author Contributions

TP: project conception, experimental work and data interpretation, and manuscript preparation. ACG and DP: experimental work and data interpretation. SW, KH, OA, and EM: experimental work. SV and AL: data interpretation. AS and LD: manuscript preparation. GEW: project conception and manuscript preparation. All authors contributed to the article and approved the submitted version.

## Conflict of Interest

The authors declare that the research was conducted in the absence of any commercial or financial relationships that could be construed as a potential conflict of interest.

## Publisher's Note

All claims expressed in this article are solely those of the authors and do not necessarily represent those of their affiliated organizations, or those of the publisher, the editors and the reviewers. Any product that may be evaluated in this article, or claim that may be made by its manufacturer, is not guaranteed or endorsed by the publisher.
