# Supplementary figures and images for: Use of X-Ray Fluorescence Microscopy for Studies on Research Models of Hepatocellular Carcinoma
Source: Front Public Health. 2021 Aug 20;9:711506. doi: 10.3389/fpubh.2021.711506 (PMC8417723; doi:10.3389/fpubh.2021.711506)

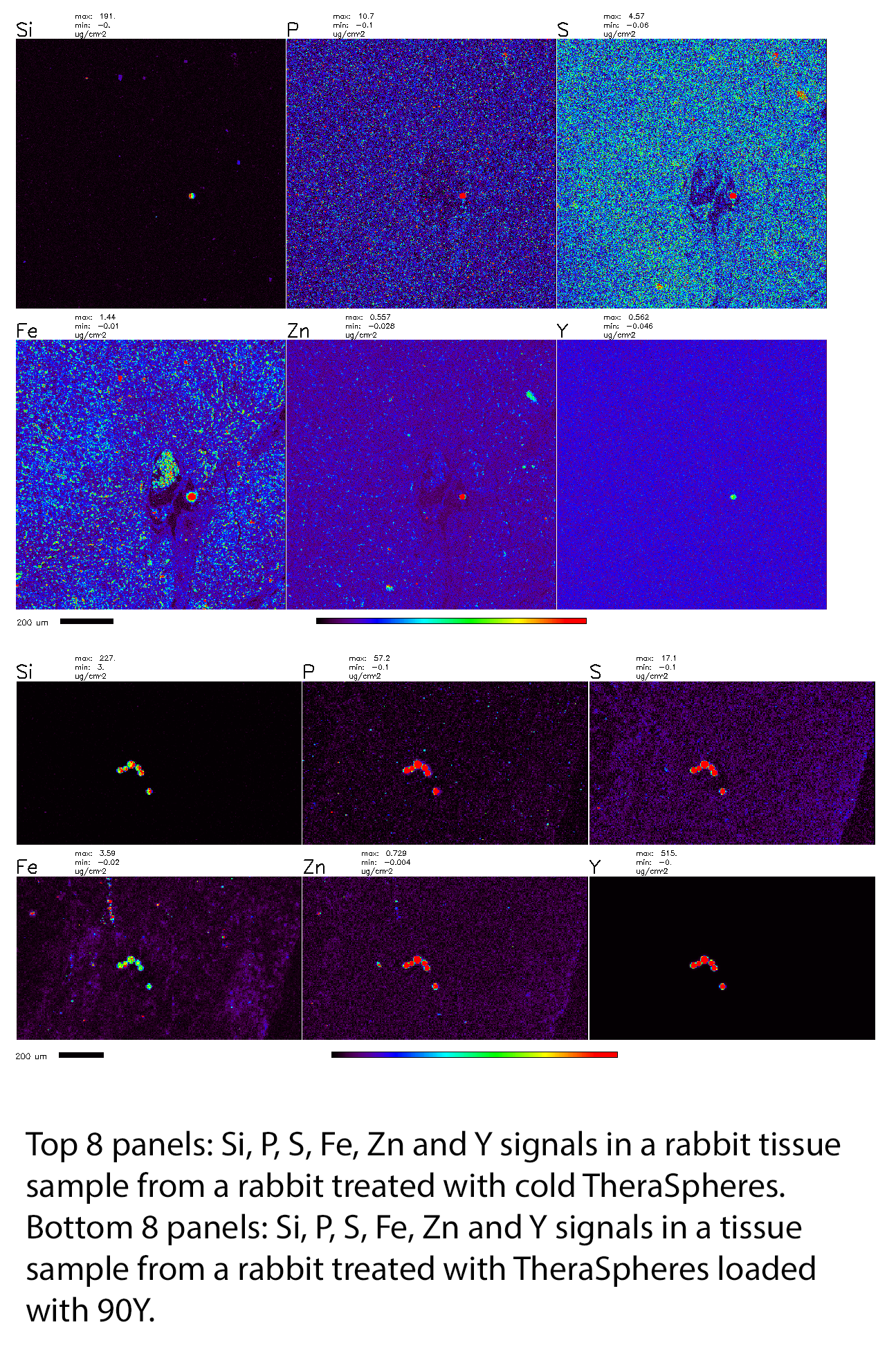

Supplement: Supplementary file 1 [file Image_1.TIF]
